# Hereditary colorectal cancer diagnostics in southern Sweden: retrospective evaluation and future considerations with emphasis on Lynch syndrome

**DOI:** 10.1007/s12687-018-0385-1

**Published:** 2018-09-24

**Authors:** Isabelle Henriksson, Karin Henriksson, Hans Ehrencrona, Samuel Gebre-Medhin

**Affiliations:** 1 Department of Clinical Genetics and Pathology, Office for Medical Services, Division of Laboratory Medicine, Lund, Sweden; 2 Division of Clinical Genetics, Department of Laboratory Medicine, Lund University, Lund, Sweden; 3 Department of Clinical Genetics and Pathology, University Hospital, SE-221 85 Lund, Sweden

**Keywords:** Hereditary, Colorectal, Cancer, Screening, Gene panel

## Abstract

Overlapping phenotypes between different hereditary colorectal cancer (CRC) syndromes together with a growing demand for cancer genetic testing and improved sequencing technology call for adjusted patient selection and adapted diagnostic routines. Here we present a retrospective evaluation of family history of cancer, laboratory diagnostic procedure, and outcome for 372 patients tested for Lynch syndrome (LS), i.e., the single most common hereditary cause of CRC. Based on number of affected family members and age at cancer diagnosis in families with genetically confirmed LS, we developed local patient selection criteria for a simplified one-step gene panel mutation screening strategy targeting also less common Mendelian CRC syndromes. Pros and cons of this strategy are discussed.

**Electronic supplementary material:**

The online version of this article (10.1007/s12687-018-0385-1) contains supplementary material, which is available to authorized users.

## Introduction

It is estimated that Mendelian predisposition to cancer is responsible for 5–10% of all colorectal cancers (CRC) (Stoffel and Boland 2015). Lynch syndrome (LS), the single most common inherited cause of CRC, shows an autosomal dominant pattern of inheritance due to germline mutations in either of the genes *MLH1*, *MSH2*, *MSH6*, *PMS2*, or *EPCAM* which eventually results in the disruption of DNA mismatch repair (MMR) in LS tumor cells (for reviews, see Kohlmann and Gruber 2004, Lynch et al. 2015). There are several other less common hereditary conditions that confer increased risk for CRC, mainly familial adenomatous polyposis (FAP or *APC*-associated polyposis caused by mutations in the *APC* gene), *MUTYH*-associated polyposis (MAP; mutations in *MUTYH*), juvenile polyposis syndrome (JPS; *BMPR1A*, *SMAD4*), *PTEN* hamartoma tumor syndrome (PHTS; *PTEN*), Peutz-Jeghers syndrome (PJS; *STK11*), and polymerase proofreading-associated polyposis (PPAP; *POLE* and *POLD1*) (for review, see Valle 2017).

In LS there is an increased risk for cancers other than CRC predominantly in the endometrium and to a lesser extent in ovaries, stomach, small bowel, urinary tract, brain, hepatobiliary tract, and skin (Kohlmann and Gruber 2004; Lynch et al. 2015; Møller et al. 2018). An affected family branch usually contains several individuals in subsequent generations with early onset LS spectrum tumors. Yet, as for all hereditary cancer syndromes the expected pattern of inheritance and clinical phenotype is sometimes obscured by limited family history data and/or incomplete disease penetrance in mutation carriers. Occasionally, the different CRC predisposition syndromes are confused due to overlapping clinical presentation (Jo et al. 2005; Aretz 2010; Spier et al. 2015; Rohlin et al. 2017).

A definitive diagnosis of LS is often obtained through a step-wise laboratory investigation including MMR functional analysis revealing DNA microsatellite instability (MSI) and/or immunohistochemical (IHC) lack of MMR protein expression in tumor tissue (often lack of both MLH1 and PMS2 or MSH2 and MSH6 as they form heterodimers) and the subsequent detection of a constitutional mutation, i.e., a pathogenic sequence variant, in any of the indicated MMR genes. This diagnostic strategy is complicated by the fact that MSI (or IHC lack of MLH1 and PMS2) is also seen in approximately 15% of sporadic CRC due to somatic biallelic methylation of the *MLH1* promoter (Aaltonen et al. 1993; Boland et al. 1998; Cunningham et al. 2001). MMR-deficiency in rectal cancer, however, is rare and should be considered an indicator of LS (Nilbert et al. 1999; de Rosa et al. 2016). MLH1/PMS2-deficiency of somatic origin can be distinguished by concomitant mutation in *BRAF*, frequently at codon 600 (V600E), which rarely occurs in LS-associated CRC. Yet, since 20–50% of CRC with somatic MLH1-deficiency do not display the *BRAF* V600E mutation, its value in the triage of patients for mutation screening is limited (Parsons et al. 2012), and *MLH1* hypermethylation-specific assays therefore need to be considered.

During the past two decades, growing knowledge and awareness about LS and other hereditary causes of CRC together with improved DNA sequencing technology have been paralleled by an increased number of referrals for genetic evaluation. For decision-making purposes to meet this demand, we reviewed previous patient referrals to our clinical genetics unit that led to any type of laboratory investigation regarding LS. We herein present the data obtained including family history of cancer and laboratory results and costs. Based on this outcome we developed local patient selection criteria for an alternative one-step laboratory diagnostic approach in which a panel of genes is screened for pathogenic mutations covering all major hereditary CRC syndromes.

## Materials and methods

### Patients and clinical data

The study was performed as part of a quality assessment project at the Department of Clinical Genetics in Lund, a unit that serves a population of approximately 1.5 million inhabitants in the southern health care region in Sweden. Local guidelines for referral of patients with early onset CRC and/or positive family history were available for health care providers (Supplementary Material 1). All referrals, i.e., 412 cases, subjected to any type of laboratory investigation regarding LS during the period of 1996–2012 were included in the study. Informed written consent for cancer genetic investigation was collected from each proband as part of the clinical routine. Forty cases were excluded from the study due to lack of data concerning clinical information and family history of cancer (nine cases), lack of tissue from a symptomatic individual (seven cases), or because a relative was already enrolled (24 cases). This resulted in a cohort of 372 adult probands. The types of laboratory investigations performed included MMR functional analyses in tumor tissue with MSI testing and/or IHC staining for any of the MMR proteins MLH1, PMS2, MSH2, and MSH6, targeted analysis of the *BRAF* V600E mutation in tumor tissue DNA (introduced in 2009), and mutation screening of one or several of the MMR genes *MLH1*, *PMS2*, *MSH2*, and *MSH6* in leukocyte DNA (sole analysis in four patients). Laboratory results, pedigrees, and data concerning tumor diagnoses in the family were retrieved from the proband’s medical record. For each pedigree, the cluster of first-degree relatives (CFDR) with the largest number of LS-associated tumors was determined, taking into account colorectal, endometrial, ovarian, gastric, small bowel, and upper urinary tract cancers. Metachronous and synchronous LS-associated tumors were counted as independent tumor cases. CFDR was defined as at least one affected individual within a single family branch. The lowest age at diagnosis (LAD) was determined for each CFDR, however, taking into account also any affected second-degree relatives in the same family branch.

### Statistical methods

The nonparametric Mann-Whitney-U test was used to test for differences in continuous variables. *P*-values of < 0.05 were considered statistically significant (two-tailed testing). All statistical analyses were performed using R version 3.2.2 (R Core Team 2015, Vienna, Austria, https://www.R-project.org/), and plots were constructed using the base and beeswarm version 0.2.1 (Aron Eklund 2015, http://CRAN.R-project.org/package=beeswarm) packages.

### Generating criteria for direct gene panel mutation screening

**Fig. 1:**
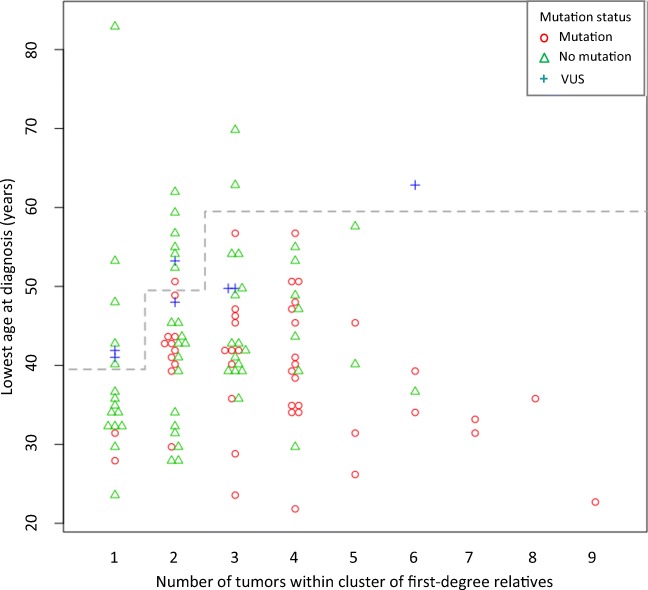
Number of LS-associated tumors in clusters of first-degree relatives (CFDR), lowest age at diagnosis (LAD), and laboratory outcome for patients subjected to MMR gene mutation screening. Each data point represents a CFDR. VUS: variant of uncertain significance. Proposed cutoff for direct gene panel mutation screening is indicated (dashed line)

A scatter plot with the number of tumors in each CFDR and LAD was generated including all patients subjected to MMR gene mutation screening; CFDR harboring a pathogenic sequence variant or a variant of uncertain significance (VUS) were indicated (Fig. 1; for description and classification of variants, see Supplementary Material 2). This scatter plot was used to define three criteria, each allowing for direct mutation screening in a simulated diagnostic approach: (a) CFDR with one tumor and LAD < 40 years, (b) CFDR with ≥ 2 tumors and LAD < 50 years, and (c) CFDR with ≥ 3 tumors and LAD < 60 years. These chosen criteria would allow the identification of all but one of the families diagnosed with LS in our cohort (Fig. 1). In addition, to comply with Swedish national guidelines which promote MMR functional testing for all patients diagnosed with CRC < 50 years, cases with a single tumor (CFDR = 1) and LAD in the range of 40–49 years would initially be selected for MMR functional analysis; cases with MSI and/or MMR protein deficiency would subsequently be offered germline MMR gene mutation screening.

### Calculation of costs

Laboratory costs were charged by external laboratories affiliated to Lund University Hospital and included extraction of DNA from blood samples, retrieval of paraffin-embedded tumor tissue from archives, laboratory analysis, data interpretation, and data reporting. Calculation of costs: all costs were converted to the levels charged in 2012 and converted from Swedish krona to euro (€). MMR functional analysis, 356 €; targeted *BRAF* V600E analysis, 640 €; germline Sanger sequencing of 1 MMR gene, 556 €; 2 genes, 1022 €; 3 genes,: 1422 €; 4 genes, 1689 €; massively parallel (gene panel) sequencing including *MLH1*, *MSH2*, *MSH6*, *PMS2*, *EPCAM*, *APC*, *MUTYH*, *BMPR1A*, *SMAD4*, *PTEN*, *STK11*, *POLE*, and *POLD1*, 1648 €.

## Results

### Outcome of LS standard laboratory process

**Fig. 2:**
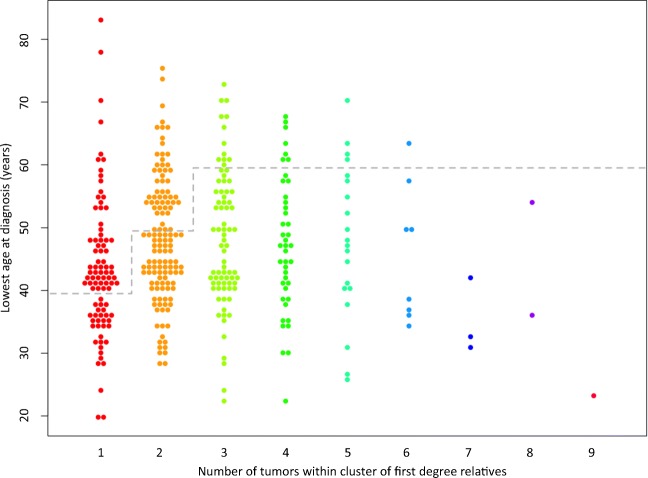
Number of LS-associated tumors in clusters of first-degree relatives (CFDR) and lowest age at diagnosis (LAD) for entire cohort. Each data point represents a CFDR. Proposed cutoff for direct gene panel mutation screening is indicated (dashed line)

**Fig. 3:**
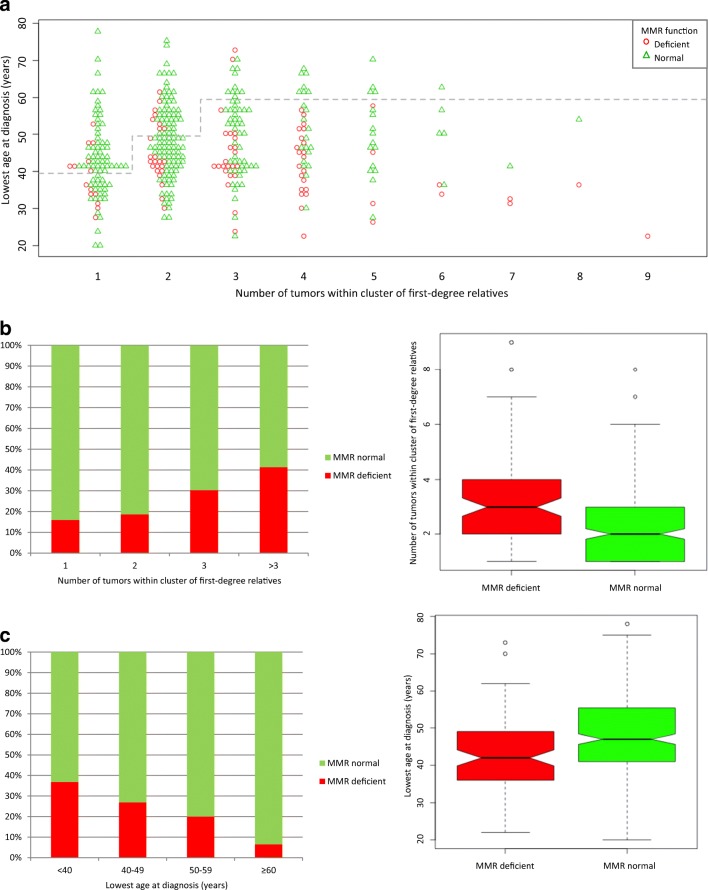
Number of LS-associated tumors in clusters of first-degree relatives (CFDR) and lowest age at diagnosis (LAD), and outcome in patients subjected to MMR functional analysis. Each data point represents a CFDR. Outcome of MMR functional analysis (n = 368); proposed cutoff for direct gene panel testing is indicated (dashed line) (**a**). Relative frequency bar graphs and notched box plots visualizing the relationship between MMR functional status and number of tumors in CFDR (**b**) or LAD (**c**), respectively

The entire cohort is shown graphically with number of tumors in CFDR and LAD in Fig. 2. The mean number of tumors in CFDR in the cohort was 2.5 and mean LAD was 47. Except for the initial study period during which number of tumors in CFDR tended to be larger, values for CFDR and LAD seemed stable over time (Supplementary Material 3a and 3b, respectively). Of the 372 patients included in the cohort, 368 patients were investigated with MMR functional analyses of which 92 patients (25%) were considered to have an MMR deficient tumor (Fig. 3a). Compared to CFDR with normal MMR function, CFDR with MMR deficiency had larger numbers of tumors (*P* = 0.00008; Fig. 3b) as well as lower LAD (*P* = 0.00002; Fig. 3c). A total of 114 patients were subjected to MMR gene mutation screening of which 48 (42%) had an LS-associated mutation (13% of the entire cohort) and another seven individuals had a VUS (Fig. 1). Almost all (47/48) patients with mutation had tumors that displayed MMR functional deficiency (one patient not investigated; Supplementary Material 2). The proportion of identified mutations was largest in *MSH2* (46%), followed by *MLH1* (31%), *MSH6* (21%), and *PMS2* (2%) (Table 1). Except for the initial study period during which the number of tumors in CFDR with mutation tended to be larger, values for CFDR and LAD seemed stable over time (Supplementary Material 3c and 3d, respectively).

**Table 1:** Proportion of pathogenic mutations, number of LS-associated tumors and lowest age at diagnosis (LAD) in clusters of first-degree relatives (CFDR) for each MMR gene

Gene	*MLH1*	*MSH2*	*MSH6*	*PMS2*
No. of patients with mutation	15 (31%)	22 (46%)	10 (21%)	1 (2%)
Mean no. of tumors in CFDR	4.3 (2–9)	3.4 (1–7)	3.2 (2–6)	2
Mean LAD in CFDR (years)	37 (23–48)	39 (22–57)	42 (34–51)	44

### Applying criteria for direct gene panel testing

If applied to our cohort, the criteria for direct gene panel testing would target 237 patients of which 31 represented a CFDR with a single tumor and LAD below 40 years, 77 a CFDR with two tumors and LAD below 50 years, and 129 a CFDR with three or more tumors and LAD below 60 years (Fig. 2). In addition, 40 patients represented a CFDR with a single tumor and had an LAD within the range of 40–49 years and would thus initially be offered MMR functional analysis only (Fig. 2); as six of these patients had an MMR deficient tumor they would subsequently be offered mutation screening accordingly (Fig. 3a).

### Estimation of total costs

The total cost for our LS standard laboratory process during 1996–2012 was 248,482 € (Table 2). The simulated total cost for direct gene panel testing and MMR functional analyses with subsequent restricted mutation screening in selected cases would be 410,948 €, i.e., a cost exceeding that of our LS standard laboratory process by approximately 65% (Table 2).

**Table 2:** Estimated costs for LS standard laboratory process versus simulated direct gene panel testing

No. of analyses	LS standard laboratory work-up			No. of analyses	Simulated approach		
	Type of analysis	Price	Total cost		Type of analysis	Price	Total cost
368	MMR functional analysis	356 €	131,008 €	237^a^	Gene panel analysis	1648 €	390,576 €
15	*BRAF* V600E	640 €	9600 €	40^b^	MMR functional analysis	356 €	14,240 €
44	Sequencing (1 gene)	556 €	24,464 €	6^c^	Sequencing (2 genes)	1022 €	6132 €
47	Sequencing (2 genes)	1022 €	48,034 €				
13	Sequencing (3 genes)	1422 €	18,486 €				
10	Sequencing (4 genes)	1689 €	16,890 €				
	Sum of total costs		248,482 €		Sum of total costs		410,948 €

^a^Cases in cohort fulfilling suggested criteria for direct gene panel testing

^b^Cases in cohort in which number of LS-associated tumor in CFDR = 1 and LAD = 40–49 years

^c^Represents cases above (^b^) with MMR deficient tumor

## Discussion

In this retrospective study, we have evaluated family history of cancer, diagnostic procedure and outcome, and laboratory costs in a cohort of patients referred for laboratory testing regarding LS. The large fraction of cases with MMR deficiency in our cohort compared to that reported in unselected CRC (25% versus 15%; Aaltonen et al. 1993, Boland et al. 1998, Cunningham et al. 2001, Bapat et al. 2009) apparently reflects an enrichment of LS in our cohort since background levels are seen when LS cases are removed (12%). The accumulation (i.e., > 15%) of MMR deficiency reported in CRC diagnosed at age ≥ 60 years due to somatic MLH1 promoter methylation (Bapat et al. 2009) was not observed in our cohort (< 7%; 3/45 cases), the discrepancy which possibly reflects the few elderly in our study. Indeed, our local guidelines encourage referrals with early onset CRC and/or positive family history (Supplementary Material 1). However, as already observed in other cohorts with early onset or familial CRC (Bapat et al. 2009; Karlitz et al. 2015), we found a positive correlation between MMR deficiency and number of LS-associated tumors as well as low LAD. Naturally, MMR deficiency, familial aggregation, and early onset disease will show significant association with LS because they are factors in determining the pathogenicity of LS gene variants, i.e., in variant classification according to The International Society for Gastrointestinal Hereditary Tumors (InSiGHT) 5-tiered scheme (Thompson et al. 2014).

Although the prevalence of LS in the Swedish population is yet to be determined, the fraction of LS detected in our cohort (13%) is well above the prevalence of 2–3% reported in unselected CRC in other Western societies (Cunningham et al. 2001; Yurgelun et al. 2017). Again, as our local guidelines support referrals of patients with positive family history and/or low age at diagnosis, the high frequency of LS in our cohort most likely reflects patient selection bias. Such bias is further supported by the mean LAD (47 years) in our cohort which is lower than that reported by The National Board of Health and Welfare in Sweden (2018) for any of the tumor types considered in the present study.

Among the 114 patients that were screened for MMR mutations in our cohort, 42% had an LS-associated mutation. Slightly higher values (53–62%) have been obtained in other Scandinavian cohorts (Lagerstedt Robinson et al. 2007; Sjursen et al. 2010). The distribution of mutations in the MMR genes in our cohort is largely similar to that recently reported in a Swedish national LS cohort, i.e., mutations in *MLH1* and *MSH2* predominate (Lagerstedt-Robinson et al. 2016).

In the present study, the chosen criteria for direct gene panel testing would target all but one of the families diagnosed with LS; this family harbors a mutation in *MSH6* and its number of LS-associated tumors (two tumors) and LAD (51 years) is the lowest and highest, respectively, among all ten families with *MSH6* mutation in our cohort. The finding is also in agreement with reports of attenuated disease penetrance and later onset of disease in *MSH6* (and *PMS2*) mutation carriers (Plaschke et al. 2004; Senter et al. 2008; Baglietto et al. 2010; Sjursen et al. 2010; Møller et al. 2018). The observed lower mean number of tumors in CFDR and higher mean LAD in families with *MSH6* or *PMS2* mutation in our cohort are, however, not statistically significant (data not shown). Nevertheless, caution should be warranted when using family history of tumors as sole selection criterion in hereditary CRC diagnostics. In this context, it should also be emphasized that the pattern of inheritance for MAP is autosomal recessive and that individuals with MAP-related CRC thus often have very few or no affected relatives. Single cases of CRC diagnosed ≥ 40 years of age caused by MAP would in fact escape detection using our proposed criteria for direct gene panel testing, again limiting the usefulness of family history of tumors alone when selecting patients for mutation screening.

In theory, if applied, the proposed criteria for direct gene panel testing would have selected a large subgroup of our cohort for molecular genetic testing, thereby potentially identifying additional cases with hereditary CRC other than LS. In practice, emerging evidence show that gene panel-based screening identifies a broad set of hereditary CRC syndromes (Chubb et al. 2015; Hansen et al. 2017; Rohlin et al. 2017; Stoffel et al. 2018). In particular, Chubb and coworkers (2015) screened a cohort of 626 patients with suspected hereditary predisposition to CRC (CFDR ≥ 2, LAD ≤ 55) with a gene panel that contained *MLH1*, *MSH2*, *MSH6*, *PMS2*, *APC*, *MUTYH*, *BMPR1A*, *SMAD4*, *POLE*, and *POLD1* with a mutation carrier yield of 10.9% for LS and, notably, 3.3% for the remaining syndromes. In the report by Stoffel and coworkers (2018) gathering 430 patients diagnosed with CRC before 50 years of age, the corresponding yield was 10.7% for LS and 4.6% for known non-LS hereditary CRC conditions (i.e., mutations in *APC*, *MUTYH*, and *SMAD4*).

The calculated laboratory cost for direct gene panel testing in our study was 65% higher than that for the LS standard laboratory process. Considering the continued decline in sequencing costs during the last decade, the cost for gene panel testing is likely to decrease. We have not evaluated the potential impact of direct gene panel testing on associated administrative aspects (personnel costs, turn-around time) and clinical procedures, and its cost-effectiveness and cost-benefit in the context of a whole hereditary colorectal cancer care package. A recent assessment of cost-utility to identify LS among cases with early onset CRC indicate that most laboratory strategies, including direct mutation testing, are cost-effective versus no testing (Snowsill et al. 2015).

Our proposed selection criteria for direct gene panel testing, now in use as guidelines at our department, were tailored in retrospect from our 1996–2012 cohort and, thus, should not be introduced in other clinical settings without independent validation. We acknowledge the continued need of MMR functional analyses, e.g., in cases with a VUS in an MMR gene, in cases with no mutation identified but a strong family history of LS-associated tumors, and in cases where tumor tissue is the only specimen available. In addition, conceivably, the anticipated introduction of universal tumor tissue screening for *BRAF*-mutations and MMR protein expression for treatment stratification purposes (Cohen et al. 2017) will alter current patient referral patterns, in particular for LS. Here, continued interdisciplinary coordination is a prerequisite to maintain diagnostic routines that allow identification of patients with constitutional predisposition for CRC.

## Electronic supplementary material

ESM 1 (DOCX 15 kb)

ESM 2 (DOCX 92 kb)

ESM 3 (DOCX 42 kb)
