# Supplementary material for: Hereditary colorectal cancer diagnostics in southern Sweden: retrospective evaluation and future considerations with emphasis on Lynch syndrome
Source: J Community Genet. 2018 Sep 24;10(2):259–66. doi: 10.1007/s12687-018-0385-1 (PMC6435770; doi:10.1007/s12687-018-0385-1)
Supplement: Supplementary file 1 — (DOCX 15 kb) [file 12687_2018_385_MOESM1_ESM.docx]

**Supplementary Material 1.** Summary of guidelines for referral of patients for cancer genetic evaluation regarding colorectal cancer (CRC) at the Department of Clinical Genetics, Lund.

Any of the following:

1. A single case of CRC diagnosed before age 50.
2. CRC or endometrial cancer (EC) in two first or second-degree relatives.
3. CRC and EC in one individual.
4. Clinical suspicion of familial adenomatous polyposis (FAP).
5. LS or FAP confirmed in a family member.
