# Supplementary material for: Hereditary colorectal cancer diagnostics in southern Sweden: retrospective evaluation and future considerations with emphasis on Lynch syndrome
Source: J Community Genet. 2018 Sep 24;10(2):259–66. doi: 10.1007/s12687-018-0385-1 (PMC6435770; doi:10.1007/s12687-018-0385-1)
Supplement: Supplementary file 2 — (DOCX 92 kb) [file 12687_2018_385_MOESM2_ESM.docx]

**Supplementary Material 2.** Description of germline MMR gene variants, their associated MMR status in tumor tissue in current study, and their classification in The International Society for Gastrointestinal Hereditary Tumours (InSiGHT) database (<https://www.insight-group.org> accessed August 1 2018). InSiGHT 5-tiered scheme (Thompson, et al., Nature genetics 2014): Class 5; pathogenic, Class 4; likely pathogenic, Class 3; uncertain, Class 2; likely not pathogenic, Class 1; Not pathogenic. (Class 5); classification made by the authors. (Class 3); initially considered as a variant of uncertain significance (VUS) by the authors and depicted as such in current study. NA; Not available. NC; Present but not classified in InSiGHT database. Letters in superscript in the table refer to the figure below (corresponds to Figure 1 in current paper) in which each VUS is identified.

| Gene | Sequence variant | Type of variant | Predicted protein change | MMR deficiency | InSiGHT  classification | No. of CFDRs with variant |
| --- | --- | --- | --- | --- | --- | --- |
| *MLH1* | [c.(?_-198)_(*193_?)del](javascript:window.location.hash%20=%20'00000001';%20return%20false) | Deletion of exon(s) | p.0? | Yes | Class 5 | 1 |
| *MLH1* | c.37G>T | Nonsense | p.(Glu13*) | Yes | Class 5 | 1 |
| *MLH1* | c.306+1G>A | Splicing aberration | p.(Lys70_Glu102del) | Yes | Class 5 | 1 |
| *MLH1* | c.307-?_545+?del | Deletion of exon(s) | p.(Ala103Valfs*9) | Yes | Class 5 | 2 |
| *MLH1* | c.350C>T | Missense | p.(Thr117Met) | Yes | Class 5 | 1 |
| *MLH1* | c.546-2A>G | Splicing effect | p.(Arg182Serfs*6) | Yes | Class 5 | 1 |
| *MLH1* | c.665del | Frameshift | p.(Asn222Metfs*7) | Yes | Class 5 | 1 |
| *MLH1* | c.1050del | Frameshift | p.(Gly351Aspfs*16) | Yes | Class 5 | 1 |
| *MLH1* | c.1219C>T | Nonsense | p.(Gln407*) | Yes | NC (Class 5) | 1 |
| *MLH1* | c.1559-?_2271+?del | Deletion of exon(s) | p.? | Yes | NC (Class 5) | 1 |
| *MLH1* | c.1667+2_1667+8delinsATTT | Splicing aberration | p.? | Yes | Class 5 | 1 |
| *MLH1* | c.1812dup | Frameshift | p.(Glu605Argfs*5) | Yes | Class 5 | 1 |
| *MLH1* | c.1852_1854del | In frame deletion | p.Lys618del | Yes | Class 5 (Class 3)^a^ | 1 |
| *MLH1* | c.1943C>T | Missense | p.(Pro648Leu) | Yes | Class 5 (Class 3)^b^ | 1 |
| *MLH1* | c.2038T>C | Missense | p.(Cys680Arg) | Yes | Class 5 (Class 3)^c^ | 1 |
| *MLH1* | c.2103+1G>A | Splicing effect | p.? | Yes | Class 5 | 1 |
| *MLH1* | c.2141G>A | Nonsense | p.(Trp714*) | Yes | Class 5 | 1 |
| *MSH2* | c.(?_-125)_(1076+1_1077-1)del | Deletion of exon(s) | p.0? | Yes | Class 5 | 1 |
| *MSH2* | c.(?_-125)_(1276+1_1277-1)del | Deletion of exon(s) | p.0? | Yes | Class 5 | 1 |
| *MSH2* | c.187del | Nonsense | p.(Val63*) | Yes | Class 5 | 1 |
| *MSH2* | c.416del | Frameshift | p.(Asn139Metfs*35) | Yes | Class 5 | 1 |
| *MSH2* | c.508C>T | Nonsense | p.(Gln170*) | Yes | Class 5 | 1 |
| *MSH2* | c.645+791_1076+4894del | Deletion of exon(s) | p.(Ile217Glufs*28) | Yes | Class 5 | 1 |
| *MSH2* | c.942+3A>T | Splicing aberration | p.? | Yes | Class 5 | 2 |
| *MSH2* | c.965G>A | Missense | p.(Gly322Asp) | No | Class 1 (Class 3)^d^ | 1 |
| *MSH2* | c.1097_1098insA | Frameshift | p.(Phe366Leufs*23) | Yes | Class 5 | 1 |
| *MSH2* | c.1147C>T | Nonsense | p.(Arg383*) | Yes | Class 5 | 1 |

Supplementary Material 2 (continued)

| Gene | Sequence variant | Type of variant | Predicted protein change | MMR deficiency | InSiGHT  classification | No. of CFDRs with variant |
| --- | --- | --- | --- | --- | --- | --- |
| *MSH2* | c.1204del | Frameshift | p.(Gln402Lysfs*10) | Yes | Class 5 | 4 |
| *MSH2* | c.1225C>T | Nonsense | p.(Gln409*) | Yes | NC (Class 5) | 1 |
| *MSH2* | c.1275A>G | Splicing aberration | p.(=, Ile411_Gly426del) | No | Class 3^e^ | 1 |
| *MSH2* | c.1587del | Frameshift | p.(Glu530Lysfs*13) | Yes | Class 5 | 3 |
| *MSH2* | c.1786_1788del | In frame deletion | p.Asn596del | Yes | Class 5 | 1 |
| *MSH2* | c.1906G>C | Missense | p.(Ala636Pro) | Yes | Class 5 | 1 |
| *MSH2* | c.2013T>A | Missense | p.(Asn671Lys) | Yes | Class 3^f^ | 1 |
| *MSH2* | c.2131C>T | Nonsense | p.(Arg711*) | Yes | Class 5 | 1 |
| *MSH2* | c.2680dup | Frameshift | p.(Met894Asnfs*5) | Yes | NC (Class 5) | 1 |
| *MSH6* | c.1407T>A | Nonsense | p.(Tyr469*) | Yes | NC (Class 5) | 1 |
| *MSH6* | c.1691C>G | Nonsense | p.(Ser564*) | Yes | NC (Class 5) | 1 |
| *MSH6* | c.2780_2781insA | Frameshift | p.(Thr928Tyrfs*7) | Yes | NC (Class 5) | 1 |
| *MSH6* | c.3103C>T | Nonsense | p.(Arg1035*) | Yes | Class 5 | 3 |
| *MSH6* | c.3226C>T | Missense | p.(Arg1076Cys) | Yes | Class 4 (Class 3)^g^ | 1 |
| *MSH6* | c.3312del | Frameshift | p.(Phe1104Leufs*11) | Yes | Class 5 | 1 |
| *MSH6* | c.3619_3620del | Frameshift | p.(His1207Phefs*7) | NA | NC (Class 5) | 1 |
| *MSH6* | c.4001+2T>C | Splicing aberration | p.(Ala1268Glyfs*6) | Yes | Class 5 | 2 |
| *PMS2* | c.736_741delinsTGTGTGTGAAG | Frameshift | p.Pro246Cysfs*3 | Yes | Class 5 | 1 |

Supplementary Material 2 (continued)
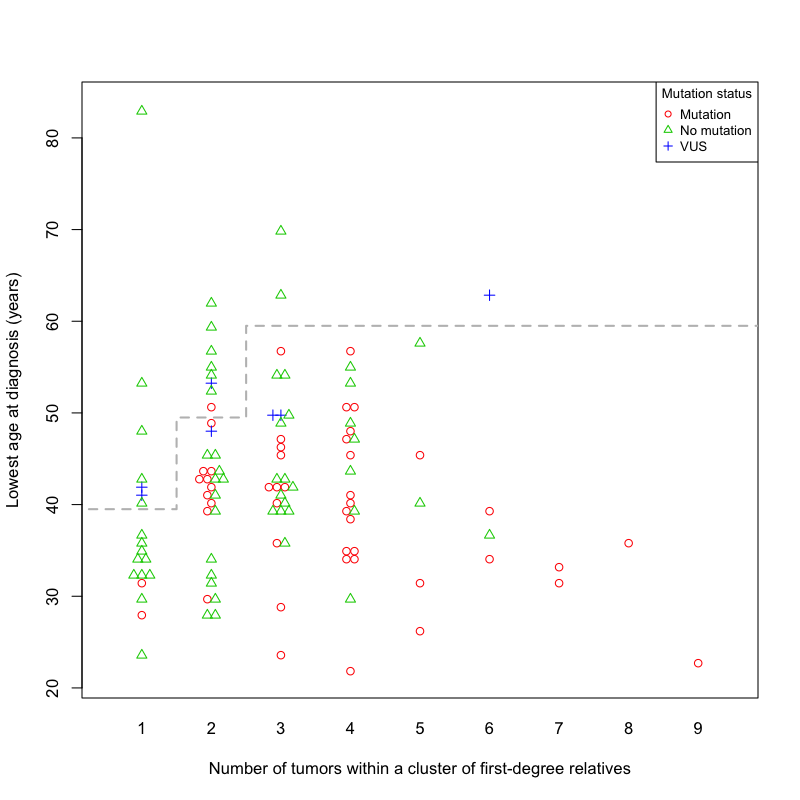


c

g

f

e

d

a

b
