# Supplementary material for: Hereditary colorectal cancer diagnostics in southern Sweden: retrospective evaluation and future considerations with emphasis on Lynch syndrome
Source: J Community Genet. 2018 Sep 24;10(2):259–66. doi: 10.1007/s12687-018-0385-1 (PMC6435770; doi:10.1007/s12687-018-0385-1)
Supplement: Supplementary file 3 — (DOCX 42 kb) [file 12687_2018_385_MOESM3_ESM.docx]

**a**

**b**

**c**

**d**
